# Supplementary figures and images for: Behavioral Economics in the Epidemiology of the COVID-19 Pandemic: Theory and Simulations
Source: Int J Environ Res Public Health. 2022 Aug 3;19(15):9557. doi: 10.3390/ijerph19159557 (PMC9368471; doi:10.3390/ijerph19159557)

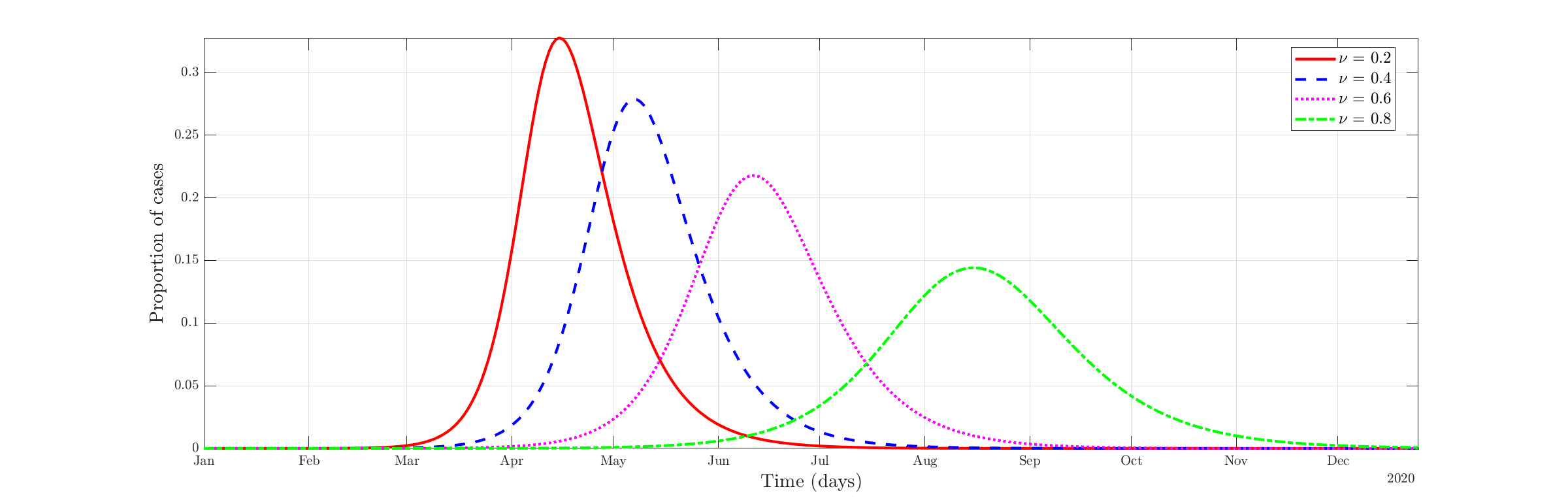

Supplement: Supplementary file 1 [file ijerph-19-09557-s001.zip › figure 1.png]

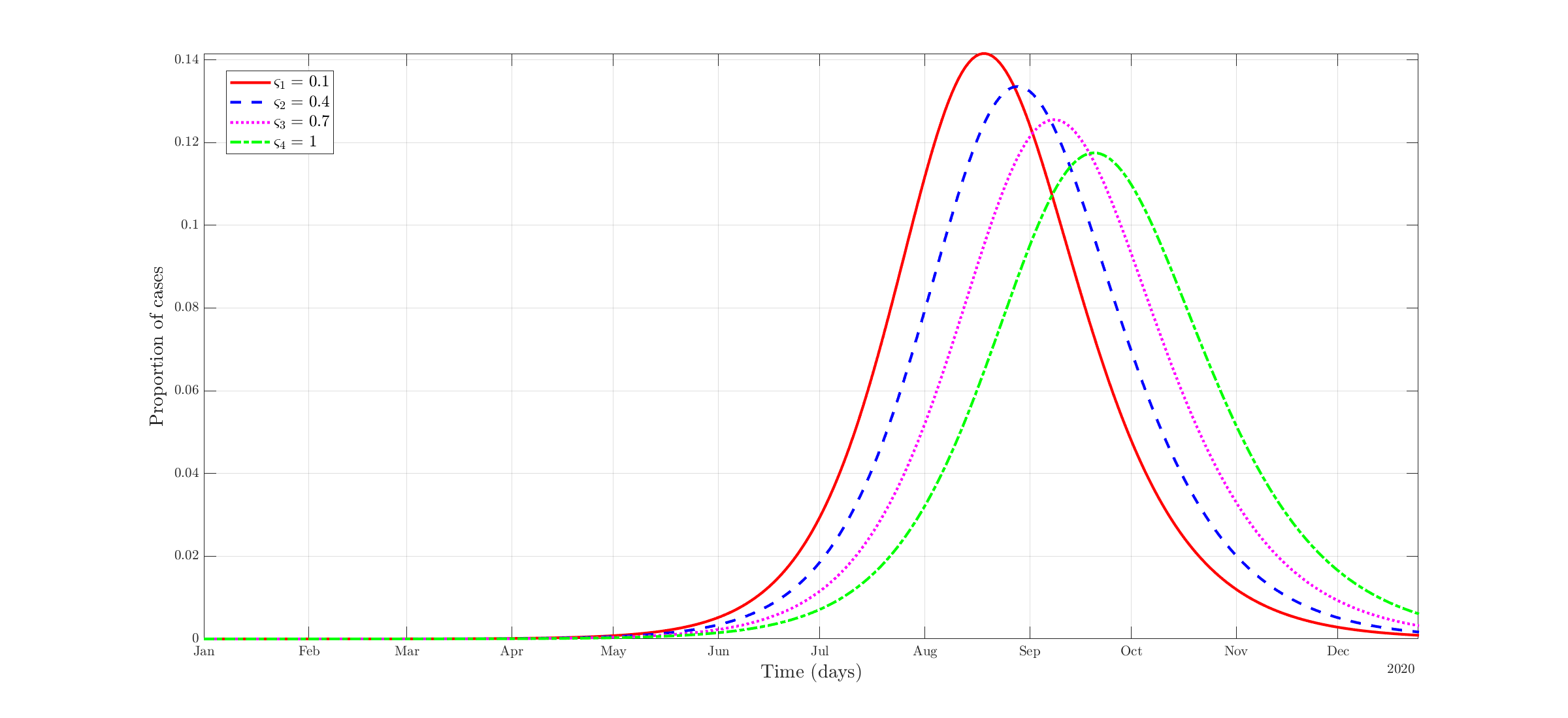

Supplement: Supplementary file 1 [file ijerph-19-09557-s001.zip › figure 2.png]

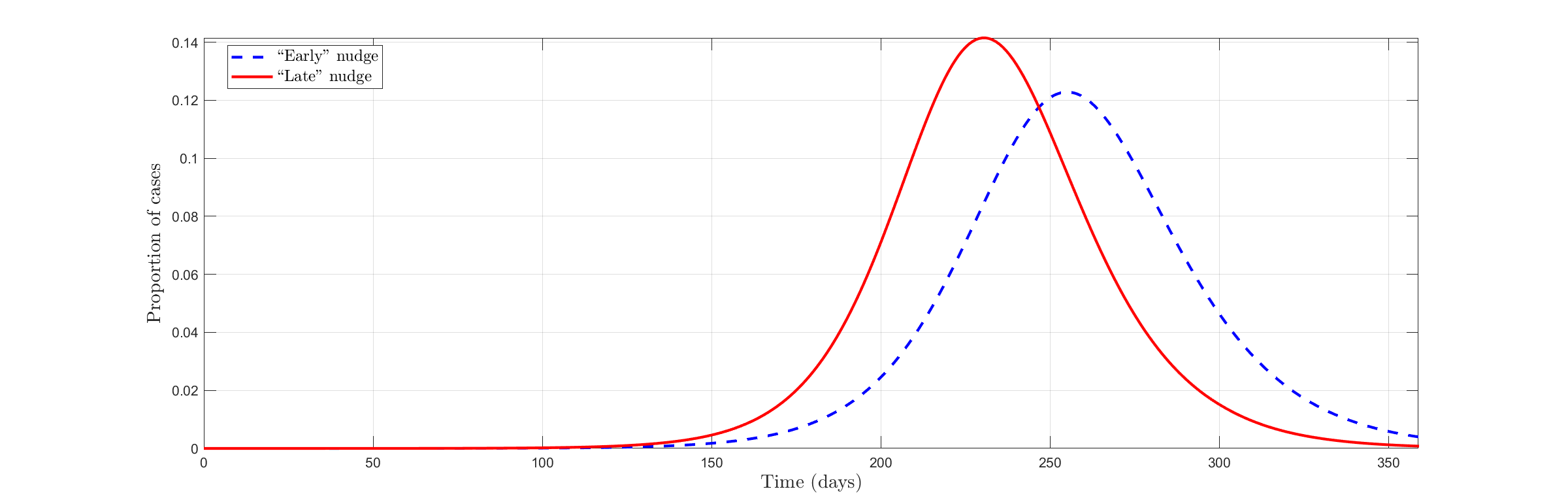

Supplement: Supplementary file 1 [file ijerph-19-09557-s001.zip › figure 3.png]

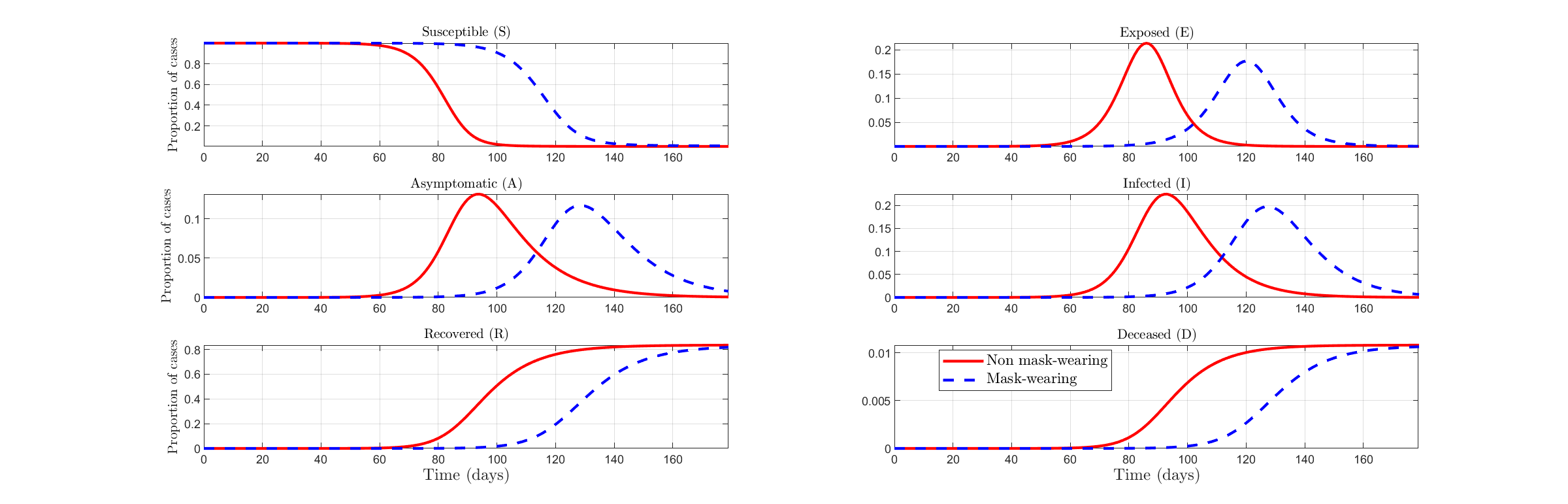

Supplement: Supplementary file 1 [file ijerph-19-09557-s001.zip › figure 4.png]

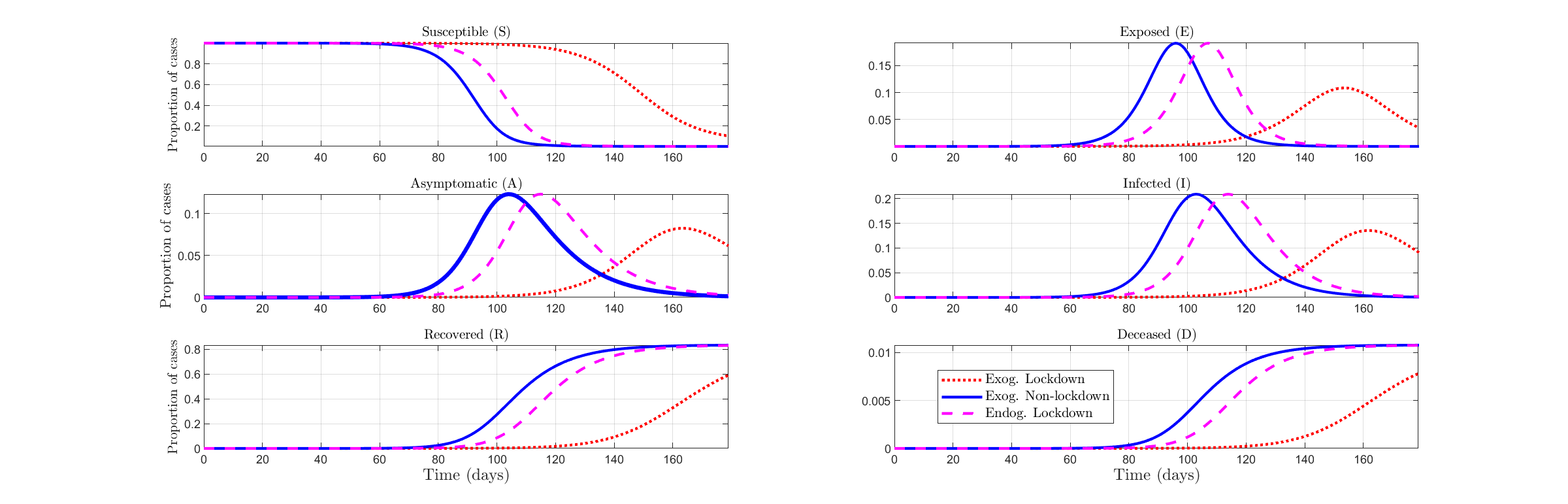

Supplement: Supplementary file 1 [file ijerph-19-09557-s001.zip › figure A1.png]

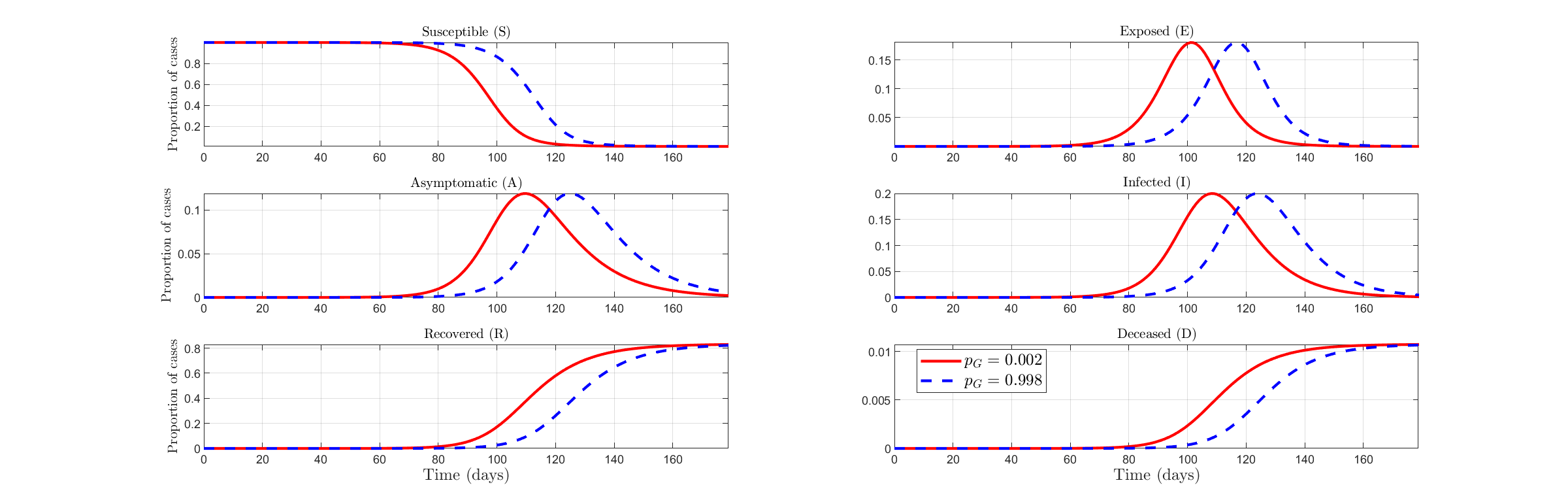

Supplement: Supplementary file 1 [file ijerph-19-09557-s001.zip › figure A2.png]

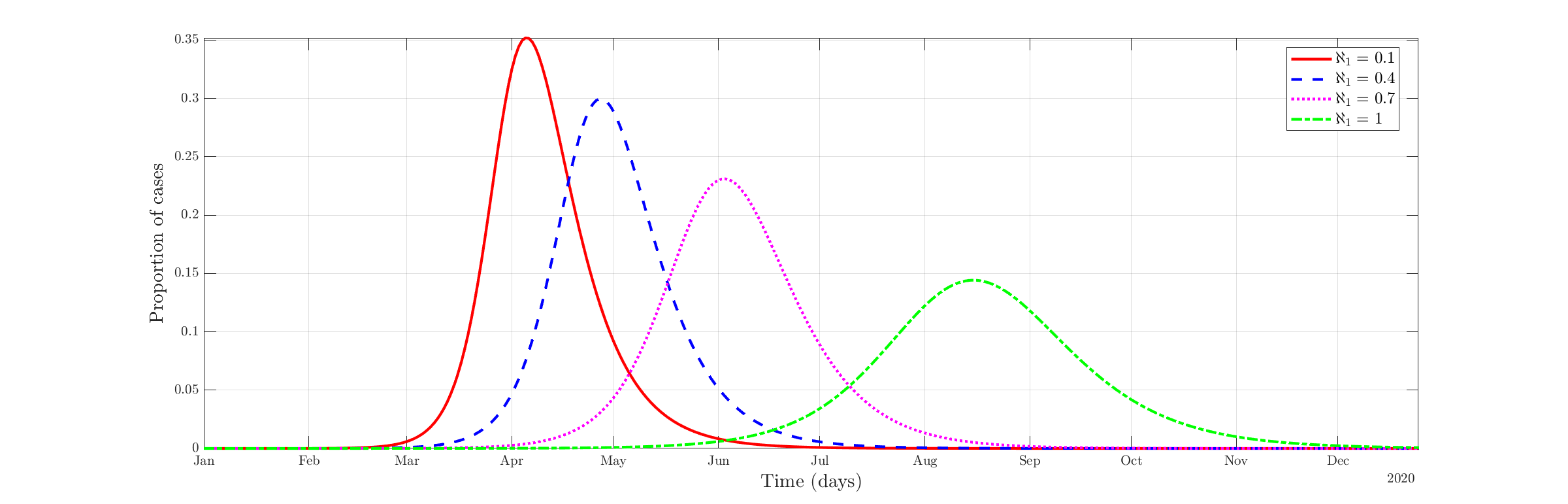

Supplement: Supplementary file 1 [file ijerph-19-09557-s001.zip › figure A3.png]

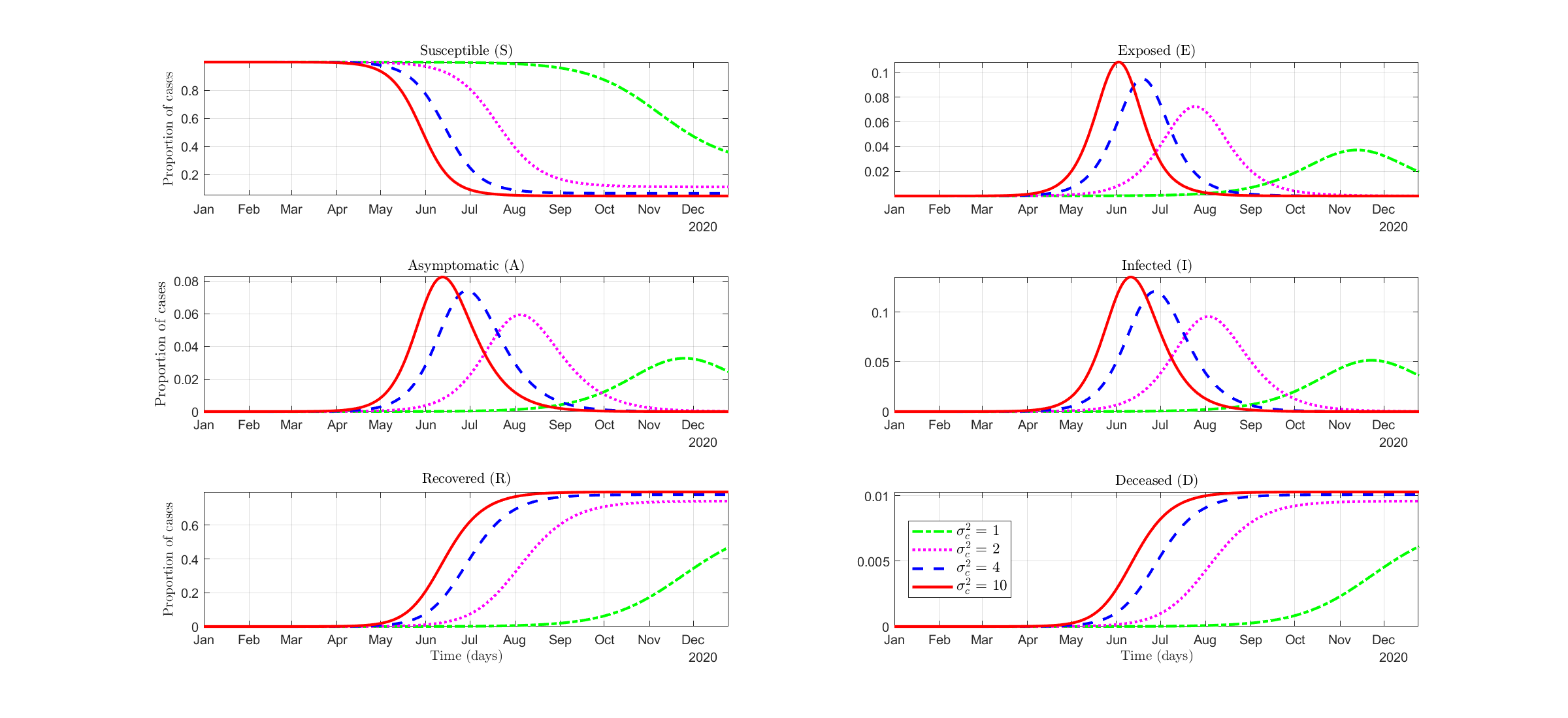

Supplement: Supplementary file 1 [file ijerph-19-09557-s001.zip › figure A5.png]

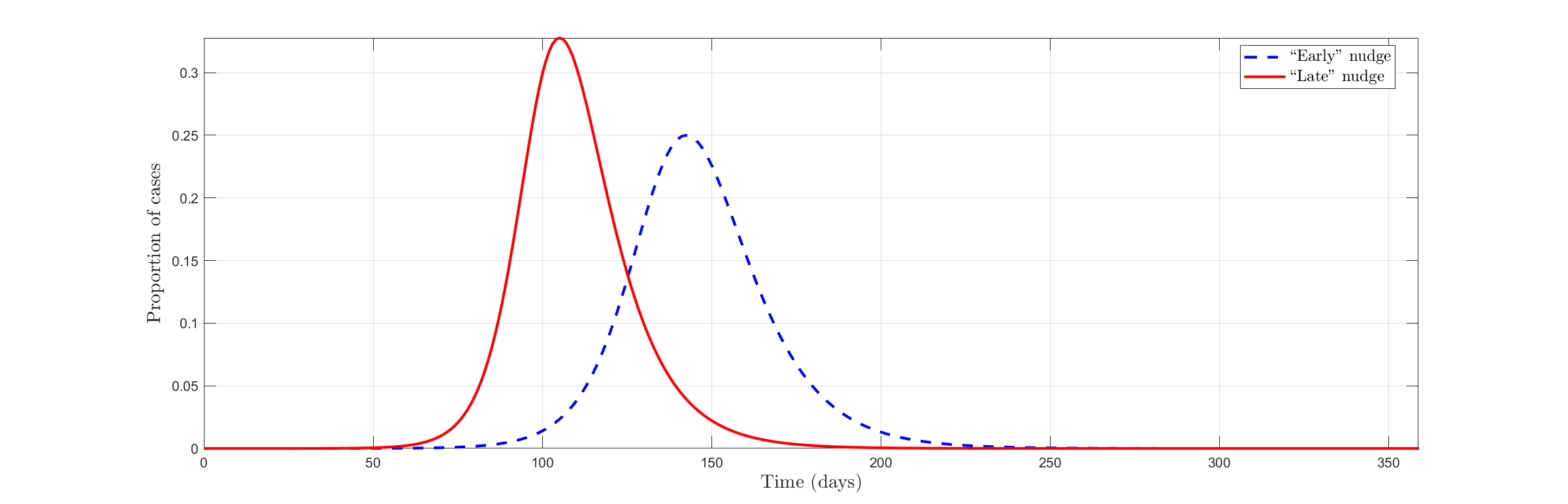

Supplement: Supplementary file 1 [file ijerph-19-09557-s001.zip › figure A6.png]

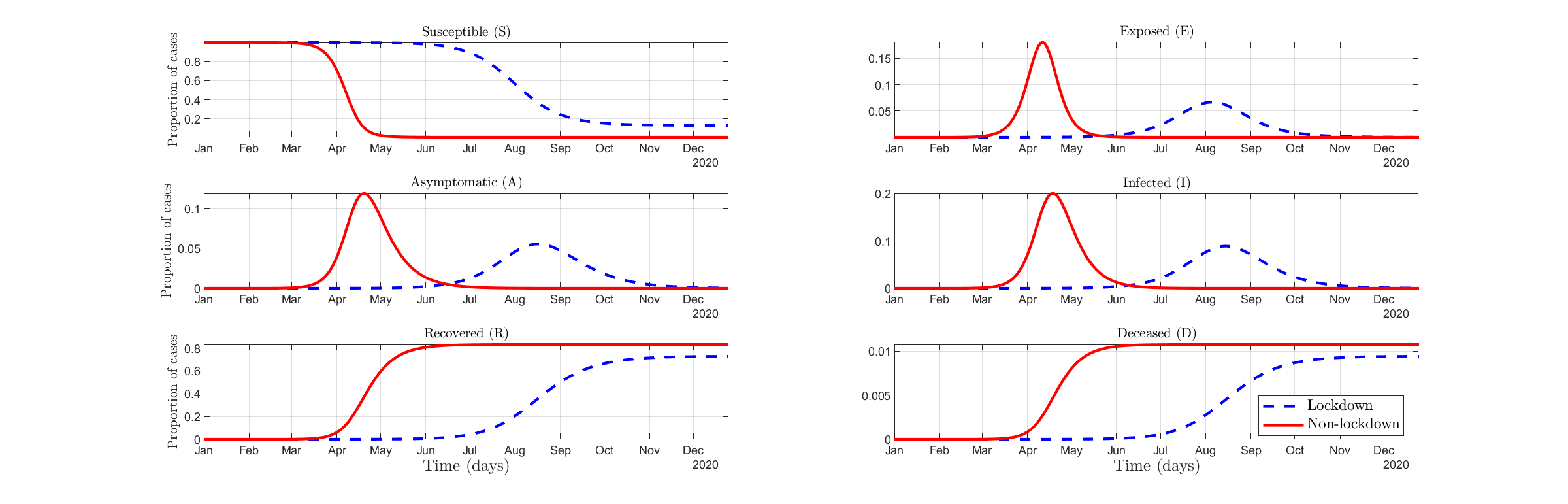

Supplement: Supplementary file 1 [file ijerph-19-09557-s001.zip › figure_A4.png]
